# Exploration of the experiences of working stressors and coping strategies associated with menstrual symptoms among nurses with shifting schedules: a Q methodology investigation

**DOI:** 10.1186/s12912-021-00759-0

**Published:** 2021-11-26

**Authors:** Shu-Chuan Yu, Hsiao-Pei Hsu, Jong-Long Guo, Shu-Fen Chen, Shu-He Huang, Yin-Chen Chen, Chiu-Mieh Huang

**Affiliations:** 1 Department of Nursing, Yonghe Cardinal Tien Hospital, Yonghe Dist., New Taipei, Taiwan; 2 Department of Nursing, College of Nursing, National Yang Ming Chiao Tung University, Taipei, Taiwan; 3 Department of Health Promotion and Health Education, College of Education, National Taiwan Normal University, Taipei, Taiwan; 4 Department of Nursing, Taipei Veterans General Hospital, Taipei, Taiwan; 5 Institute of Clinical Nursing, College of Nursing, National Yang Ming Chiao Tung University, 155, Sec.2, Li-Nong Street, 11221 Taipei, Taiwan

**Keywords:** Female fertility cycle, Workload, Healthcare

## Abstract

**Background:**

The essence and workload of nursing can easily lead to burdens associated with female nurses’ menstrual symptoms, and consequently, result in decreased working performance. Without effective support this can lead to resignation due to maladaptation. This study adopted Q methodology to explore the experience of working stressors and coping strategies associated with menstrual symptoms among nurses with shifting schedules.

**Methods:**

Data were collected in two stages. First, in-depth interviews were conducted to collect nurses’ experiences. Sentences that best fit the study’s purpose were extracted for the construction of Q statements. Second, nurses were allowed to subjectively rank these Q statements by using Q-sorts. A total of 90 participants ranked the designed Q statements. The Q factor analysis revealed a five-factor solution that accounted for 48.90% of the total variance.

**Results:**

The five evident factors included: menstrual symptoms interfering in collaboration with colleagues, deficiency of professional function and stress due to symptoms burden, diverse experiences without a clear pattern, adapted self-management with and without medication use, and stress due to symptoms burden and using medication for self-management.

**Conclusions:**

The identification of these five groups may facilitate the development of responsive strategies to meet nurses’ preferences. Furthermore, identifying workplace factors that are associated with the adverse effects of menstrual symptoms on nurses will be helpful for nursing supervisors and hospital managers. Additionally, strategies that can be implemented to create supportive work environments are discussed.

## Background

Trouble-free menstrual cycles are considered an indicator of reproductive health [1]. However, menstrual discomfort, a common gynecological complaint among women, results in cyclical symptoms burden. Large-scale research indicates that most women (74%) suffer from menstrual symptoms [2]. Repeat occurrences of menstrual symptoms can significantly disrupt women’s daily life and social functioning, reduce their abililty to perform work-related tasks, and decrease their quality of life [3–5]. A large scale study of Japanese females estimated the annual economic burden of menstrual symptoms as approximately US$8.6 billion [6]. A systematic review revealed that the annual cost of direct and indirect treatment for menstrual symptoms was $12–36 billion [7]. Within one year after the diagnosis of primary or secondary dysmenorrhea, the average total medical expenditure is $1,916 US and $2,465 US, respectively [8].

Previous studies suggest an association between shift work and menstrual function [9]. Shift work is prevalent among nurses who work days, evenings, nights, or rotating shifts. Approximately 40% of nurses have a fixed, long-term night, schedule or worked night shifts [10]. Studies examining the relationship between shift work and menstrual cycle characteristics among nurses revealed that the prevalence rate of dysmenorrhea was 70.7%. Among them, 91.2% worked in rotating shifts [11]. 35% of nurses experience menstrual cycle irregularity [12]. The prevalence rates of dysmenorrhea (58%) and cycle irregularity (53%) increased after working nights [13]. In addition to dysmenorrhea and cycle irregularity, shift work is associated with changes in menstrual blood volume and length of the menstrual cycles (< 21 days or > 35 days) [13–15].

Menstrual symptoms are associated with work-related stress [16], and occupational stress increases physiological arousal during workdays in contrast to off-days [17, 18]. Nursing care involves both physically and emotionally demanding work [10]. A national-wide survey revealed that menstrual pain was significantly associated with control at the workplace, social support from coworkers, and job security [16]. Some scholars propose that changes in menstrual function may influence shift workers’ intolerance toward working demands [13].

Discussing the experience of menstrual symptoms may still be considered a social taboo and frowned upon [19]. Women may fear being viewed as weak, and thus, hesitate to express their discomfort or request assistance while experiencing menstrual symptoms [20]. This explorative study used Q methodology to elicit subjective experience and identify the shared experience patterns among shift-working nurses regarding their work-related stressors and strategies for coping with menstrual symptoms. We intended to cluster nurses based on the patterns that were associated with their experiences of working while experiencing menstrual discomfort. By clustering nurses with similar experiences into groups, group-responsive workplace support can be derived, rather than universal approaches for all nurses. The results will be helpful in establishing supportive strategies to improve wellbeing among nurses with shift schedules and for nursing supervisors and hospital managers to identify workplace factors that are associated with adverse impacts of menstrual symptoms on nurses.

## Methods

### Study design and participants

The Q methodology was used in this study to investigate the various common experiences among nurses with shifting schedules. Since experiences may vary according to the workplace culture and infrastructure at different hospitals, we enrolled eligible participants from a medical center, a regional hospital, and a district hospital to obtain the nurses’ experiences across hospitals at various levels. Ninety clinical staff members were recruited from the three hospitals and 30 nurses from each hospital, respectively. The nurses who met the following criteria were included in the study: were currently employed and had at least 1 year of clinical experience, had worked night shift schedules in the past 3 months, had experienced menstrual symptoms in the past 3 months, and were willing to participate in the study and provide written informed consent.

### Measures

Characteristics of nursing staff background were collected via questionnaire and included types of hospital, education, marital status, pregnancy experience, level of nurse career advancement, age, and total years of clinical experience. The questionnaire regarding menstrual symptoms was adapted from the Cyclic Pelvic Pain and Discomforts symptoms cluster suggested by the Association of Women’s Health, Obstetric and Neonatal Nurses [21]. Symptoms were grouped as pelvic pain (four items), perimenstrual physical discomfort (seven items), and perimenstrual mood discomfort (nine items). Participants indicated the level of severity of each symptom, within the previous month, on a 5-point Likert scale ranging from 0 (never) to 4 (extremely severe).

### Q statements

To explore the shared experience patterns among shift-working nurses, Q statements were developed by gathering statements through face-to-face interviews. The interviews were conducted by the first author. During the interviews, the interviewer introduced herself and then explained the purpose of the study and interview questions. The participants were asked open-ended questions about their experiences of menstrual symptoms in relation to their work-related stressors and how they coped with menstrual discomfort. Interviews lasted approximately 20–30 min, were audiotaped and, transcribed verbatim. We obtained data saturation after 15 nurses were interviewed.

We developed sentences which closely described their experiences. The primary categories were extracted from the transcripts. Five categories of shift working experience regarding their work-related stressors and the strategies they coping with menstrual discomforts were derived (Table 1). The categories were: deficiency of professional function, menstrual symptoms burden, interference in collaborating with colleagues, self-management coping, and colleague supports. The two subcategories under deficiency of professional function were patient care and professional responsibility. Under menstrual symptoms burden, the three subcategories were physical burden, cognitive burden, and psychological burden. The two subcategories under self-management coping were medication use and no medication use. There were no subcategories under interference in collaborating with colleagues and working environment supports. Researchers reviewed relevant texts and generated a list of Q statements under each category. Five experts and scholars from related specialties were invited to examine the representative Q statements. The content validity index (CVI) was used to evaluate the Q statements. Only statements with CVI values >0.8 were retained for further Q sorting [22]. A modified list of 47 Q statements was obtained by clarifying the semantics of individual statements and eliminating duplicate statements (Table 1).

**Table 1:** List of the Q statements

Category of Q statements		Items of Q statements
**A. Deficiency of professional function**		
	¬ **Patient care**	I cannot concentrate and frequently am distracted during work.
		Even if I have to take a leave because of menstrual symptoms, I still worry about my patients.
		I cannot take good care of my patients.
		I cannot ‘abandon my patients’ due to my responsibility.
		Patients or family members may consider me impatient due to my tone and this affects my relationship with patients.
	¬ **Professional responsibility**	I may neglect some details during my work.
		I cannot rest peacefully due to my professional responsibility.
		It is perceived negatively if I take a rest in nursing station.
		I cannot take leave because of my professional responsibility.
		Due to menstrual symptoms, I lost my enthusiasm for nursing care.
**B. Menstrual symptoms burden**		
	¬ **Physical burden**	Workload, stress, and prolonged standing or walking, can worsen my menstrual symptoms.
		Due to menstrual symptoms, my efficiency during work worsened.
		Due to menstrual symptoms, I feel easily tired.
		Due to menstrual symptoms, I have become less responsive.
	¬ **Cognitive burden**	Due to menstrual symptoms, I have no patience for anything.
		I always regret being impatient easily.
		Due to menstrual symptoms, I seldom think.
	¬ **Psychological burden**	Due to menstrual symptoms, my mood is unstable.
		Due to menstrual symptoms, I am easily irritable.
		Due to menstrual symptoms, I have a negative mood towards my family members.
**C. Interfering collaboration with colleagues**		
		If I take leave due to menstrual symptoms, it will cause trouble for my head nurse.
		Taking menstrual leave may result in a bad impression by my head nurse and will affect my credit or promotion.
		I worry my poor efficiency in work may affect my occupational and interpersonal relationships.
		I worry I may be labeled and considered to be exaggerating or a fake.
		I worry my colleagues’ workload may increase if I take leave.
		I worry my colleagues will consider me difficult to work with due to my persistent menstrual symptoms.
		My unit is short on manpower, there is no way I can take a menstrual leave.
		I am unwell physically and no colleague is available to replace/ assist me.
		Colleagues did not take leave due to menstrual symptoms, so I will not either.
**D. Self-management coping**		
	¬ **With medication use**	I have pain-killers on hand in case of need.
		I took pain-killers to control my menstrual symptoms.
		I will take pain-killers beforehand so I can maintain my efficiency in work.
		I worry about developing dependence for pain-killers.
		When I anticipate a greater workload, I will increase my dose of pain-killers.
	¬ **Without medication use**	I finish my work as soon as possible so as to back to nursing station for rest.
		I use hot packs or special postures to lessen menstrual symptoms.
		I prepare myself with self-encouragement if I anticipate menstrual symptoms will occur.
		I reduce talking to maintain physical strength.
		I use willpower to tolerate menstrual symptoms until my work is done.
		The pain is intermittent, I tolerate pain by distracting my attention.
		I want to lie in bed to reducing the discomfort of menstrual symptoms.
**E. Colleague support**		
		My colleagues are empathic and can accept me.
		Colleagues will volunteer to help when I suffer from menstrual pain.
		The atmosphere in my working environment indicate understanding the condition of menstruation induced physical discomfort.
		When I request assistance, colleagues are always willing to help.
		Colleagues help me by sharing the heavy work so I can rest for a while.
		I owe my colleague a favor for replacing/assisting me.

### Procedure of data collection

**Fig. 1:**
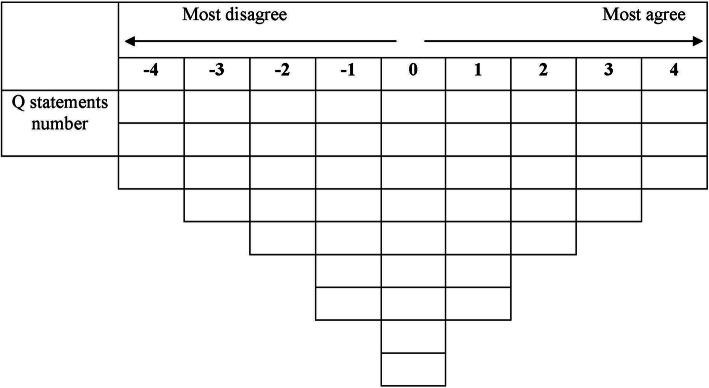
A Q-sort gird for rank-ordering Q statements.

Study protocol was approved by the institutional review boards of the participating hospitals. Upon ethics approval, the research team visited potential participants at the hospitals and arranged appropriate times to demonstrate the Q-sort procedure. Participants were given individual account names and passwords to perform the Q sorting online. A total of 90 participants ranked the Q statements. They were asked, “Are the following statements congruent with your experience?” The nurses ranked the degree of congruence between these Q statements and their experiences of work-related stressors and the strategies they used to cope with menstrual symptoms. Their opinions on the Q statements were entered by forced choice in a Q-sort grid (Fig. 1), generating a quasi-normal distribution of degrees of agreement (+4)/disagreement (-4) with the statements. A Q-sort example of the user interface was reported in our previous study [23]. The participants selected from flexible time options to log in to complete the Q-sort.

### Data analysis

The Q method adapts a type of exploratory factor analysis to cluster participants who performed Q sorts into groups. The Q sorts data were entered into PQ Method version 2.35 [24] and Q factor analysis was performed using principal components analysis with varimax rotation. Factors were identified using the Q factor analysis. This process produced a factor array and derived composite Q sorts which represented experience patterns associated with working stressors and coping strategies related to menstrual symptoms among nurses with shifting schedules. The characterizing statements, ranked at the most positive ends of the composite Q-sort of each factor (i.e., +3 and +4), were used to describe the experience of nurses who loaded significantly onto the given factor.

## Results

Ninety clinical nurses, aged 20 to 48 years (mean = 30.56, ± standard deviation 7.30 years), were included in this study. The average clinical experience of the participants was 9.28 ± 7.22 years. Most participants were unmarried (*n* = 66, 73.30%), and had a college degree or higher (*n* =59, 65.5%).

To determine the number of retained factors, a combination of eigenvalues and a screen plot was used. The Q factor analysis revealed a five-factor solution that accounted for 48.90% of the total variance. The explained variances of the five factors were 17.47%, 10.14%, 8.46%, 6.56%, and 6.27%, respectively. We used the term “groups” to indicate the factors resulting from the factor analysis because Q factor analysis generated clusters of nurses with similar experiences. The initial description is presented according to the characterizing statements that ranked at the most positive ends (i.e., +3 and +4) of the composite Q-sort of each group.

### Group 1: menstrual symptoms interfering in collaboration with colleagues

Participants associated with group 1 emphasized that experiencing menstrual symptoms interfered in their collaboration with colleagues. They worried that their absence might increase their colleagues’ workload (+3); could not take menstrual leave due to a shortage of manpower (+4); their colleagues could not provide help if they felt unwell (+4); and hesitated to take leave if their colleagues did not (+3). They had pain-killers on-hand as needed (+4) and lessened their symptoms by lying in bed (+3). They also easily felt tired (+3). Twelve participants significantly loaded in this group (Table 2).

**Table 2:** Characterizing statements of the five groups: the statements ranked at the most positive ends (+3, +4)

Categories of Q Statements	Group 1	Group 2	Group 3	Group 4	Group 5
A: Deficiency of professional function		I cannot concentrate and frequently am distracted during work.(+4) I cannot take good care of my patients.(+3) I may neglect some details during my work.(+3)	I cannot concentrate and frequently am distracted during work.(+3)		I may neglect some details during my work.(+4)
B: Menstrual symptoms burden	Due to menstrual symptoms, I feel easily tired.(+3)	Workload, stress, and prolonged standing or walking, can worsen my menstrual symptoms.(+3) Due to menstrual symptoms, my efficiency during work worsened.(+4) Due to menstrual symptoms, I feel easily tired.(+3) Due to menstrual symptoms, I have become less responsive.(+4)	Workload, stress, and prolonged standing or walking, can worsen my menstrual symptoms.(+4) Due to menstrual symptoms, my efficiency during work worsened.(+3) Due to menstrual symptoms, I feel easily tired.(+4)	Workload, stress, and prolonged standing or walking, can worsen my menstrual symptoms.(+3) Due to menstrual symptoms, I feel easily tired.(+4)	Workload, stress, and prolonged standing or walking, can worsen my menstrual symptoms.(+4) Due to menstrual symptoms, I have no patience for anything.(+3) Due to menstrual symptoms, I have become less responsive.(+3)
C: Interfering collaboration with colleagues	My unit is short on manpower, there is no way I can take a menstrual leave.(+4) I owe my colleague a favor for replacing/assisting me.(+4) I worry my colleagues’ workload may increase if I take leave.(+3) Colleagues did not take leave due to menstrual symptoms, so I will not either.(+3)			I worry my colleagues’ workload may increase if I take leave.(+3)	
D: Self-management coping	I have pain-killers on hand in case of need.(+4) I want to lie in bed to reducing the discomfort of menstrual symptoms.(+3)		I want to lie in bed to reducing the discomfort of menstrual symptoms.(+4) I use hot packs or special postures to lessen menstrual symptoms.(+3)	I have pain-killers on hand in case of need.(+4) I will take pain-killers beforehand so I can maintain my efficiency in work.(+3) I finish my work as soon as possible so as to back to nursing station for rest.(+3) I use hot packs or special postures to lessen menstrual symptoms.(+4)	I have pain-killers on hand in case of need.(+4) I took pain-killers to control my menstrual symptoms.(+3) I will take pain-killers beforehand so I can maintain my efficiency in work.(+3)
E: Colleague support			My colleagues are empathic and can accept me.(+3)		

Compared with other participants, those associated with group 1 had a relatively long clinical experience (11.58 years), with an average age of 33.50 years. Most were employed at a regional hospital (60%), and 83.30% had an education level equivalent to a baccalaureate degree, 25.0% had a higher level of nursing competency (N3 and N4). Only 25.0% were married (Table 3).

**Table 3:** Background information of the five groups

	Group 1		Group 2		Group 3			Group 4				Group 5		
Variables	*n*=12		*n*=21		*n*=14			*n*=14				*n*=12		
	n	%	n	%	n	%		n	%			n	%	
Type of hospital														
Medical center	5	41.7	6	28.6	6	42.9		4	28.6			7	43.8	
Regional hospital	6	50.0	5	23.8	6	42.9		4	28.6			4	25.0	
District hospital	1	8.3	10	47.6	2	14.2		6	42.8			5	31.2	
Education level														
College	2	16.7	9	42.9	6	42.9		6	42.9			2	16.7	
Baccalaureate	10	83.3	11	52.4	8	57.1		6	42.9			10	83.3	
Graduate	0	0.0	1	4.8	0	0.0		2	14.3			0	0.0	
Marital status														
Married	3	25.0	6	28.6	3	21.4		3	21.4			4	25.0	
Other	9	75.0	15	71.4	11	78.6		9	78.6			12	75.0	
Level of nurse career advancement														
N0	0	0.0	0	0.0	3	21.4		2	14.3			0	0.0	
N1	2	16.7	6	28.6	4	28.6		4	28.6			5	31.2	
N2	7	58.3	9	42.9	5	35.7		5	35.7			7	43.8	
N3,4	3	25.0	6	28.6	2	14.2		3	21.4			4	25.0	
	mean ±	SD	mean ±	SD	mean±	SD		mean±		SD		mean±		SD
Age	33.50±	6.27	31.19±	8.08	29.71±	7.32	32.07±		9.48		32.00±		6.06	
Clinical experience	11.58±	6.76	9.38±	7.63	7.86±	7.43	9.27±		8.96		9.19±		6.68	
Menstrual symptom														
Cyclic pelvic pain	7.67±	2.99	8.24±	2.77	5.64±	1.73	6.57±		1.91		7.88±		2.68	
Perimenstrual physical discomfort	12.25±	3.76	14.48±	5.40	10.21±	4.37	11.93±		5.13		11.75±		5.38	
Perimenstrual mood discomfort	12.33±	6.65	15.00±	7.19	8.50±	5.89		8.71±	6.76			12.94±		7.75

### Group 2: deficiency of professional function and stress due to symptoms burden

Participants whose perspectives were consistent with those of group 2 emphasized their experience of a deficiency of professional function and stress due to symptoms burden. They were unable to concentrate during work (+4); unable to provide optimal care to patients (+3); and easily became negligent regarding details (+3). During work, their menstrual symptoms worsened (+3); efficiency worsened (+4); they easily became tired (+3); and became less responsive (+4). Twenty-one participants significantly loaded on this factor (Table 2).

The average age of the participants associated with group 2 was 31.19 years, with an average clinical experience of 9.38 years. Most were employed at a district hospital (47.6%), and 52.4% had an education level equivalent to a baccalaureate degree: 28.6% had a higher level of nursing competency (N3 and N4), and 28.6% of the participants were married (Table 3).

Regarding symptoms’ severity, the group 2 nurses had the highest scores for clinical pelvic pain (8.24), perimenstrual physical discomfort (14.48), and perimenstrual mood discomfort (15.00) (Table 3).

### Group 3: diverse experience with no clear pattern

The experiences of those in group 3 fell into various categories without a clear pattern. Among the five categories of Q statements, group 3 evident experiences covered four categories, only the category of interfering collaboration with colleagues was not included. However, of the five groups, group 3 was the only one with evident experiences that was associated with the category of working environment support: 14 participants significantly loaded on this factor. These nurses were concerned about not concentrating during work (+3), symptoms burden (worsening menstrual symptoms during work: +4, less efficient during work: +3, easily tired: +4), self-management coping (hot-packs or special posture: +3, lying in bed: +4), and empathy from and acceptance by colleagues (+3). Fourteen participants loaded significantly on this factor (Table 2).

Among all the participants, those associated with group 3 were relatively young (29.71 years), with an average clinical experience of 7.86 years. Most of the participants were employed at medical centers (42.9%) and regional hospitals (42.9%): 57.1% of the participants had an education level equivalent to a baccalaureate degree. 14.2% had a higher level of nursing competency (N3 and N4): 21.4% were married (Table 3).

Regarding symptoms’ severity, participants in group 3 had the lowest scores for clinical pelvic pain (5.64), perimenstrual physical discomfort (10.21), and perimenstrual mood discomfort (8.50) (Table 3).

### Group 4: adapted self-management with and without medication use

Nurses who shared experiences that were associated with group 4 primarily emphasized their experience by using self-management coping. They had pain-killers on hand as needed (+4) and also took analgesics beforehand (+3). They pushed themselves to finish work as soon as possible (+3) and use hot-packs and special postures to lessen symptoms (+3). During work, their menstrual discomfort worsened (+3) and they easily felt tired (+4). They also worried that their absence might increase their colleagues’ workload (+3). Fourteen participants loaded significantly on this factor (Table 2).

The average age of the participants associated with group 4 was 32.07 years, with an average clinical experience of 9.57 years. Most were employed at a district hospital (42.9%), and 42.9% of the participants had an education level equivalent to a baccalaureate degree: 21.4% had a higher level of nursing competency (N3 and N4) and 21.4% were married (Table 3).

### Group 5: stress due to symptoms burden and using medication for self-management

Nurses who shared experiences that were associated with group 5 equally emphasized symptoms burden and self-management coping. It is worth noting that medication use was the major self-management coping. They had pain-killers on hand as needed (+4); took over-the-counter medicine (+3), and took analgesics beforehand (+3). During work, their menstrual symptoms worsened (+4); they became impatient (+3), and less responsive (+3). They also easily became negligent (+4). Sixteen participants significantly loaded on this group (Table 2).

The average age of the participants associated with group 5 was 32.00 years, with an average clinical experience of 9.19 years. Most were employed at a medical center (43.8%), and 93.8% had an education level equivalent to a baccalaureate degree: 25.0% had a higher level of nursing competency (N3 and N4). Additionally, 25.0% were married (Table 3).

## Discussion

This study intended to explore experiences associated with working stressors and coping strategies related to menstrual symptoms among shift-working nurses. Q factor analysis revealed five groups of nurses with different patterns of evident experiences. As expected, menstrual symptoms burden was the most evident experience among this particular population: shift-working nurses. Among the five groups of nurses, four groups emphasized experiences of menstrual symptoms burden, especially physical burdens. The prevalence of menstrual symptoms is high both among women [25] and nurses [26]. Night work and physically demanding work are related to menstrual disturbance [1]. The combination of menstrual symptoms with shift-work and high-intensity work may amplify, the overall pressure on nurses. Our findings suggested that shift-working nurses emphasized physical symptoms burden more than psychological symptoms burden. This highlighted the necessity to address their physical need at the workplace.

In contrast, the least evident experience across the five groups of nurses was support from their colleagues. Only nurses in group 3 reported receiving colleagues’ assistance while suffering from menstrual pain. This finding implies that there was a lack of support from colleagues. However, expressing menstrual symptoms may be viewed negatively. Women preferred to not express that they were suffering from menstrual symptoms, due to the fear of being viewed as weak or difficult [20]. Additionally, a large scale, online survey that was conducted via social media (i.e., Facebook and Twitter) revealed that only 20.1% of women who took sick leave from work or school due to menstrual symptoms told their employer or school the real reason. Most of the women reported that while providing the reason for their sick leave they did not mention menstrual symptoms or made up another reason, while some reported that they did not provide a reason. [19]. Similarly, nurses on duty may feel embarrassed to inform their colleagues that they are suffering from menstrual symptoms. Thus, they do not receive assistance since their colleagues are unaware of their needs.

The nurses associated with group 1 shared the evident experience of concerns related to interference in collaboration with colleagues. Among the five groups, members of group 1 were relatively older with an average clinical experiences of 11.58 years. These findings suggest that nurses of group 1 constrained themselves and hesitated to request assistance, despite having over 10 years of clinical experience. The most emphasized experiences were associated with limited manpower, such as being unable to take menstrual leave and lacking colleagues who are available to provide assistance. A survey study revealed that 82% of nursing staff were aware of the availability of menstrual leave, and 97% of them agreed that it is necessary. However, only 8.4% have actually used the leave in the past six months [11]. The nurses in group 1 were the only ones with evident experiences associated with colleague collaboration and less evident experiences associated with symptoms burden. While addressing the needs of this group, colleagues’ opinions should be taken into consideration, especially their concerns regarding sharing the workload.

The nurses who loaded on group 2 emphasized both deficiency of professional function and stress due to menstrual symptoms burden. Compared to the other groups, nurses in group 2 emphasized their deficiency in professional function in addition to menstrual symptoms burden. A sense of responsibility is critical for nursing, given the positive association between a sense of professional responsibility and quality of patient care [27]. The menstrual symptoms’ severity of the nurses in group 2 was also relatively high. These results are congruent with previous findings of a negative association between symptoms’ severity and patient care [28]. Other studies also observed an association between professional responsibility and menstrual symptoms’ severity [29, 30]. For nurses in group 2 who had relatively severe menstrual symptoms, support for relief from physical symptoms would help reduce deficiency of professional function.

The nurses in group 3 were associated with experiences that fell into various categories without a clear pattern. This is the only group with an evident experience related to support from colleagues, in terms of receiving voluntary help from them. Group 3 adopted self-management strategies without medication to relive physical symptoms, including using hot packs and postural changes (e.g., lying in bed) to relieve menstrual symptoms. The results were similar to those of a previous study indicating that nurses may do things such as drink warm water, use hot packs, or lie down and rest to manage the symptoms [11]. However, another study reported that some nurses choose to use medication (i.e., analgesics) for symptom relief [31]. When providing support for those in group 3, non-medication-based pain management strategies should be considered.

Most of the women who encountered menstrual symptoms attempted manage it themselves instead of visiting a physician [32]. Analgesic use is a common strategy for managing menstrual pain [33]. The nurses in group 4 shared the evident experiences of emphasizing self-management. Unlike those in group 3 who avoided medication use, those in group 4 attempted to manage their symptoms both with and without medication. Alternatively, group 5 emphasized the experiences of using medication for symptom management. Group 5 appeared to have a strong tendency toward relying on analgesics to relieve symptoms. Living with pain is not only affects physical health but also has impacts on several aspects of life, such as work, social functioning, and family [34]. Individuals with chronic pain may experience job loss as a result of their pain symptoms. In addition, menstrual dysfunction was suggested as an indicator of shift workers’ intolerance toward working demands [13], and commensurately, intolerance was manifested by menstrual symptoms. Therefore, support related to the working demands is needed in addition to support for symptom management.

### Implementation of practices

The evident experience of each group was identified using Q methodology. According to the Q sorting procedure, nurses weighed their degree of agreement with each statement in relation to other statements. Rather than evaluating each statement separately on a Likert-type scale, the nurses were expected to prioritize all of the statements simultaneously, allowing personally evident experiences to be captured. Responsive approaches, according to each group, may be beneficial for attaining more effective workplace support. Tailored strategies responding to the needs of nurses in each group can enhance appropriate assistance.

Our findings suggest that some nurses who had an average of over 10 years of clinical experience were concerned about how menstrual symptoms interfered with collaboration with colleagues. When there are shortages in nursing manpower, colleagues may rely on experienced nurses for assistance or problem solving related to patient care/administrative tasks. To provide effective support, supervisors and administrators should consider these concerns associated with colleagues’ interactions. For nurses in group 2 who have relatively severe menstrual symptoms, support should be focused on relieving physical symptoms, such as providing an appropriate space to rest and allowing short breaks. It was evident in this study that nurses’ physical needs in the workplace must be addressed. Nurses who suffered from menstrual symptoms may not need to request sick leave if their workplace allows short breaks and provides appropriate space for them to rest. Supportive strategies related to caring for the burdens associated with physical symptoms will help reduce deficiency of professional function. Some nurses who had relatively low severity of symptoms utilized coping strategies that did not include medication (group 3). Providing an appropriate space to rest and access to tools that can help with symptom management (i.e., a warm drink and hot packs) will be helpful support strategies. The major difference between nurses in groups 4 and 5 was that nurses in group 5 primarily relied on analgesics for symptoms relief. Support for this group in terms of education regarding the adverse effects of analgesic use should be provided, especially for those who are long-term users. Additionally, menstrual symptoms may manifest in shift workers’ intolerance toward working demands. Support only for symptoms management may not be sufficient for nurses in groups 4 and 5. Reevaluating work demands will help prevent nurses from resigning. Further, our findings imply that there is a lack of support from colleagues; this is may be due to manpower shortages or because nurses do not inform their colleagues that they are suffering from menstrual symptoms as they feel embarrassed to do so. Addressing the stigma surrounding menstrual symptoms can help create an open and supportive work environment.

## Limitations

This study has several limitations. The primary limitation is that nurses’ experiences may vary according to their workplace culture and the infrastructure at different hospitals. Although we enrolled eligible participants from three hospitals to extend heterogeneity, a cross cultural study will increase the generalizability of the results. Second, the nonrandom sampling method that was used in this study may have reduced the applicability of our findings to shift-working nurses with menstrual symptoms. However, the Q methodology is useful for clustering individuals with similar experiences rather than exploring the prevalence of experiences.

## Conclusions

This study used the Q methodology to identify nurses’ experiences related to work-related stressors and coping strategies associated with menstrual symptoms among nurses with shifting schedules. The identification of the five groups can facilitate the development of responsive strategies to meet nurses’ preferences. The five groups of experiences of menstrual symptoms were interfering collaboration with colleagues, deficiency of professional function and stress due to symptoms burden, diverse experience with no clear pattern, adapted self-management with and without medication use, and stress due to symptoms burden and using medication for self-management. Grouping nurses based on similar experiences may enable the identification of their needs and thereby, help provide them with appropriate workplace assistance.

## Data Availability

The datasets used and/or analyzed during the current study are available from the corresponding author on reasonable request.

## Abbreviations

CVI: content validity index
